# Relationship of systemic IL-10 levels with proinflammatory cytokine responsiveness and lung function in agriculture workers

**DOI:** 10.1186/s12931-018-0875-z

**Published:** 2018-09-03

**Authors:** Tricia D. LeVan, Debra J. Romberger, Mohammad Siahpush, Brandon L. Grimm, Athena K. Ramos, Patrik L. Johansson, Tzeyu L. Michaud, Art J. Heires, Todd A. Wyatt, Jill A. Poole

**Affiliations:** 1 College of Public Health, University of Nebraska Medical Center, Omaha, NE USA; 2 Veterans Affairs Nebraska Western Iowa Healthcare System, Omaha, NE USA; 3 Department of Internal Medicine, University of Nebraska Medical Center, Omaha, NE USA; 4 Department of Environmental, Agricultural and Occupational Health, University of Nebraska Medical Center, Omaha, NE USA; 5 Department of Epidemiology, University of Nebraska Medical Center, Omaha, NE USA

**Keywords:** Agriculture, IL-10, Organic dust, Airway disease

## Abstract

**Background:**

Agriculture workers are exposed to microbial component- and particulate matter-enriched organic dust aerosols. Whereas it is clear that exposure to these aerosols can lead to lung inflammation, it is not known how inflammatory responses are resolved in some individuals while others develop chronic lung disease. Interleukin (IL)-10 is an immunomodulatory cytokine that is recognized as a potent anti-inflammatory and pro-resolving factor. The objective of this study was to determine whether there is a relationship of systemic IL-10 and proinflammatory responses and/or respiratory health effects in humans with prior agriculture exposure.

**Methods:**

This is a cross sectional study of 625 veterans with > 2 years of farming experience. Whole blood was stimulated with or without organic dust and measured for IL-6, TNFα and IL-10. Participants underwent spirometry and respiratory symptoms were assessed by questionnaire.

**Results:**

We found that baseline IL-10 concentration from the whole blood assay was inversely associated with ΔTNF-α (*r* = − 0.63) and ΔIL-6 (*r* = − 0.37) levels. Results remained highly significant in the linear regression model after adjusting for age, sex, BMI, race, education, smoking status, and white blood cell count (ΔTNF-α, *p* < 0.0001; ΔIL-6, *p* < 0.0001). We found no association between chronic cough (*p* = 0.18), chronic phlegm (*p* = 0.31) and chronic bronchitis (*p* = 0.06) and baseline IL-10 levels using univariate logistic regression models. However, we did find that higher FEV_1_/FVC was significantly associated with increased baseline IL-10 concentration.

**Conclusions:**

Collectively, these studies support a potential role for IL-10 in modulating an inflammatory response and lung function in agriculture-exposed persons.

## Background

Interleukin (IL)-10 is an immunomodulatory cytokine that is recognized as a potent anti-inflammatory and pro-resolving factor in many model systems [1, 2]. IL-10 is secreted by various cell types including T cells, B cells, macrophages, mast cells, eosinophils, and dendritic cells [3]. The systemic and local IL-10 response has been shown to be of pathophysiological relevance in several malignancies, infectious diseases, autoimmune diseases, and atopic disorders [1, 4]. IL-10 can work as a counter regulatory cytokine as in the setting of lipopolysaccharide (LPS)-induced sepsis whereby LPS-induced IL-10 can inhibit expression of proinflammatory cytokines and also inhibit signaling of pattern recognition receptors [4]. As one identified mechanism, IL-10 activates STAT3 in macrophages and T cells to transcriptionally repress and suppress proinflammatory responses [4–6]. In animal studies, absence of IL-10 greatly increases murine susceptibility to experimental endotoxemia [7]. In the murine lung environment, IL-10 reduces subepithelial fibrosis associated with chronically inhaled endotoxin [8], promotes resolution of neutrophilic lung inflammation [2], and reduces viral and bacterial-induced lung immunopathology [9, 10].

Organic dust in the agricultural environment is enriched in an abundance and diversity of gram-positive and gram-negative bacterial components and particulate matter [11–13]. Chronic inhalation of these complex dusts can result in adverse respiratory health consequences including asthma, chronic bronchitis, and chronic obstructive pulmonary disease (COPD) [14]. The airway inflammatory consequences associated with agriculture exposures consist of neutrophil and lymphocyte influx, increased IL-17 responses, airway hyper-responsiveness, proinflammatory cytokine release, and lung function decline over time [14–17]. There is evidence that IL-10 might play a role in agriculture exposure-associated lung disease. Administration of exogenous IL-10 reduced acute grain dust-induced airway hyper-responsiveness and neutrophil influx in mice [18]. It has also been shown that rodents repetitively exposed to organic dust extracts display a Th1/Th17 microenvironment and influx of exudative/activated macrophages [19, 20]. This is important because IL-10 is a recognized inhibitor of Th17 and activated macrophage responses, particularly in a cytokine milieu rich in tumor necrosis factor (TNF)-α [4].

It is not known whether there is a relationship of systemic IL-10 and proinflammatory responses and/or respiratory health effects in humans with prior agriculture exposure, which was the objective of this study. We hypothesized that high baseline systemic IL-10 levels would be inversely associated with proinflammatory cytokine response and adverse respiratory health indices. To study this hypothesis, we utilized cross-sectional data from a well-characterized veteran population of agricultural workers from the Midwestern United States to determine whether systemic IL-10 levels were associated with whole blood TNF-α and IL-6 hyper-responsiveness and indicators of respiratory health including lung function and reported symptoms.

## Methods

### Study population

A cross-sectional study of United States Veterans with agriculture exposures were recruited at the Omaha Veterans Affairs Medical Center and has been described previously [21]. During an outpatient clinic visit, the veteran was asked, “Have you worked on a farm for more than two years?” Those who answered “yes” and were between the ages of 40 and 80 years were eligible for this study. Furthermore, eligible subjects had to have no history of asthma, lung cancer, metastatic cancer to the lungs, or interstitial lung diseases such as pulmonary fibrosis, sarcoidosis or hypersensitivity pneumonitis, based upon self-report and medical chart confirmation. Those with a history of an infection or exacerbation of disease within the previous 3 weeks were excluded. Recruitment began March of 2008 and continued through December of 2013. The study was approved by the VA Institutional Review Board, and all subjects signed a written informed consent document before enrollment.

### Clinical assessments

Demographic information, years worked on a farm, smoking status and respiratory symptoms were obtained by in-person and telephone questionnaires. Ever smokers were defined as having smoked more than 100 cigarettes in their lifetime. Chronic bronchitis was defined by the American Thoracic Society guidelines as having chronic cough and chronic phlegm for three consecutive months for at least 2 years [22]. All subjects underwent spirometry, with post-bronchodilator spirometry (0.083% albuterol) performed on individuals with a forced expiratory volume in 1 s (FEV_1)_ / forced vital capacity (FVC) ratio < 0.70. COPD was defined by the Global Initiative for Chronic Obstructive Lung Disease (GOLD) classification criteria (FEV_1_/FVC < 0.7) [23]. The highest recorded FEV_1_ and FVC were used to derive height-, weight-, age-, gender-, and ethnic-adjusted values based on National Health and Nutrition Survey (NHANESIII) reference equations. [24].

### Organic dust extract (ODE)

Organic dust was collected from a swine confined animal feeding operation and prepared as previously described [25]. Briefly, settled surface dust was extracted in phosphate buffered saline, centrifuged and filter sterilized (100% ODE). The dust extract was diluted to a final concentration of 1% (vol/vol) and used in the whole blood assay.

### Whole blood cytokine assay

Blood was obtained by venipuncture and used for a cell differential and the whole blood assay [26]. Heparinized blood samples were processed within 2 h of collection. Blood was diluted at a 1:1 ratio with L-glutamine-RPMI 1640 medium (Thermo Fisher Scientific, Waltham, MA) then stimulated with ODE (1%) or sterile phosphate buffered saline. Dilute blood was incubated for 24 h at 37 °C with 5% CO_2_, and then centrifuged at 500 x g for 5 min. Cell-free supernates were stored at − 80 °C.

### TNF-α, IL-6 and IL-10 ELISAs

For IL-6 and TNF-α measurement, a sandwich ELISA was employed [26]. In brief, purified (goat) anti-human IL-6 or (mouse) anti-human TNF-α antibody (both from R&D Systems, Minneapolis, MN) were used to coat a microtiter plate overnight. Whole blood assay supernates were incubated in the wells at room temperature followed by (rabbit) anti-human IL-6 antibody (Sigma-Aldrich, St. Louis, MO) or biotinylated (goat) anti-human TNF-α (R&D Systems). Human serum-absorbed peroxidase conjugated (goat) anti-rabbit IgG (Rockland Immunochemicals, Pottstown, PA) or streptavidin-HRP for TNF-α (R&D Systems) were used for detection. The colorimetric conversion of substrate (TMB, R&D systems) was quantified at 450 nm using the VERSAmax microplate reader (Molecular Devices, San Jose, CA). Cytokine concentrations were interpolated from an integrated 8-point standard curve created using purified recombinant human proteins. The limits of detectability for the human cytokine assays were: IL-6, 60 pg/mL and TNF-α, 15 pg/mL. Human IL-10 was measured using a commercially available kit with limits of detection at 31.3 pg/mL (human IL-10 Duoset, R&D Systems).

### Statistical analyses

Study population characteristics were summarized using descriptive statistics such as mean ± standard deviation (SD) and n (%). Due to the skewed nature of IL-6, TNF-α and IL-10 levels, all values were transformed to the natural logarithm scale. Cytokine responsiveness to ODE was calculated as the difference between baseline and ODE-stimulated cytokine production. Associations between ΔLn IL-6 and ΔLn TNF-α levels and baseline Ln IL-10 were examined using Pearson’s correlation. Multivariable linear regression models were used to examine ΔLn IL-6 and ΔLn TNF-α as predictors of Ln IL-10 level, while adjusting for age (continuous variable), sex (male/female), body mass index (BMI kg/m^2^, < 25, 25–29.9, ≥ 30), race (white/other), education (< high school, ≥ high school), smoking status (never, former, current) and white blood cell count. The association between chronic respiratory symptoms (cough, phlegm and bronchitis) and Ln IL-10 concentration was determined using Student’s t-test and multivariable logistic regression (adjustment for age, sex (male/female), body mass index (BMI kg/m^2^, < 25, 25–29.9, ≥ 30), race (white/other), education (< high school, ≥ high school), smoking status (never, former, current) and white blood cell count (10^3^/μl, continuous variable). Associations between lung function and ln IL-10 (quartiles) concentration were determined by ANOVA. Due to the non-linear nature of the ANOVA results, lung function variables (FEV_1_/FVC ratio; FEV_1_, % predicted; FVC, % predicted) were classified into four levels based on quartiles and multivariable models were examined using multinominal logistic regression. Lung function quartile was treated as the nominal response variable and the model was adjusted for age (continuous variable), sex (male/female), body mass index (BMI kg/m^2^, < 25, 25–29.9, ≥ 30), race (white/other), education (< high school, ≥ high school), smoking status (never, former, current) and white blood cell count (1 × 10^3^/μl, continuous variable). Corresponding OR and 95% CI of increased lung function (higher quartile relative to lower quartile) associated with Ln IL-10 concentration (continuous), were calculated.

Multivariable models were generated using backwards stepwise regression analysis to identify common sociodemographic and disease-related factors independently associated with Ln IL-10, respiratory symptoms and lung function. Given their significance in other studies [27], race, sex and education were factored into the model.

## Results

There were a total of 625 individuals included in the analyses. Characteristics of participants are summarized in Table 1. Reflecting demographic trends in the VA population nationally [28], participants were predominantly older men (mean age 64.8 years; 97.9% male) who were white (96.1%) and smoked during their lifetime (80.2%). Based on our inclusion criteria, all participants had worked on a farm during their adult life, for an average of 26.5 years and the majority having worked with livestock (87.3%) and crops (81.9%). Approximately 30% of the population reported they had respiratory symptoms (chronic cough, 34.1%; chronic phlegm, 34.6%; chronic bronchitis, 24%) and 40.0% had COPD, defined as FEV_1_/FVC ratio < 0.7.

**Table 1:** Study Population Characteristics Stratified by Airway Obstruction

	Total *N* = 625
Age, Mean yrs. (SD)	64.8 (8.3)
Sex, n (%)	
Male	612 (97.9)
Female	13 (2.1)
BMI, kg/m^2^, n (%)	
< 25	91 (14.6)
25–29.9	182 (29.1)
≥ 30	352 (56.3)
Race, n (%)	
White	599 (96.1)
Other	24 (3.9)
Education, n (%)^a^	
≤ High School	266 (44.2)
> High School	336 (55.8)
Smoking Status, n (%)	
Never	124 (19.8)
Former	368 (58.9)
Current	133 (21.3)
COPD	250 (40.0)
Chronic Cough^a^	204 (34.1)
Chronic Phlegm^a^	206 (34.6)
Chronic Bronchitis^a^	142 (24.0)
Lifetime Exposure to Livestock^a^	527 (87.3)
Lifetime Exposure to Crops^a^	534 (81.9)
Worked on a Farm, Mean yrs. (SD)^a^	26.5 (20.9)
White Blood Cell Count × 10^3^/μl (SD)^a^	7.4 (2.1)
Neutrophils^a^	4.6 (1.9)
Monocytes^a^	0.68 (0.35)
Eosinophils^a^	0.23 (0.20)

*Abbreviations and Definitions: BMI* body mass index, *COPD* chronic obstructive pulmonary disease, defined as FEV_1_/FVC < 0.7; Livestock, hogs, beef and dairy cattle, and poultry; Crops, corn, sorghum, wheat, oats, and hay

^a^Numbers do not add up to 100% due to missing data

**Fig. 1:**
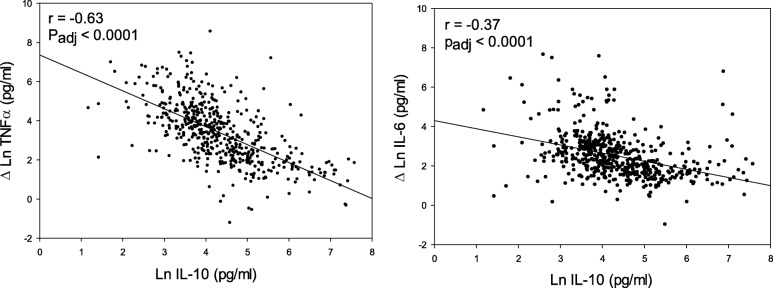
Baseline IL-10 concentration is inversely associated with responsiveness to organic dust extract. Left: x-axis is baseline level of IL-10 in the whole blood assay and y-axis is the difference between baseline and organic dust-stimulated TNF-α (Δ Ln TNF-α; *n* = 501). Right: x-axis is baseline level of IL-10 in the whole blood assay and y-axis is the difference between baseline and organic dust-stimulated IL-6 in the whole blood assay (*n* = 501). Multivariable linear regression models were adjusted for age, sex, BMI, race, education, smoking status (never, former, current), and white blood cell count. r = Pearson’s correlation coefficient

The whole blood assay was used to determine if cytokine responsiveness to ODE was correlated with IL-10 concentration. We found that baseline IL-10 concentration was inversely associated with ΔTNF-α and ΔIL-6 levels (Fig. 1). Pearson’s correlation coefficients were *r* = − 0.63 for ΔTNF-α and *r* = − 0.37 for ΔIL-6. Results remained highly significant in the linear regression model even after adjusting for age, sex, BMI, race, education, smoking status (never, former, current), and white blood cell count (ΔTNF-α, *p* < 0.0001; ΔIL-6, *p* < 0.0001). We found in the sensitivity analyses that the correlation between baseline IL-10 and ΔTNF-α and ΔIL-6 levels remained significant when stratifying by COPD status (COPD, *p* < 0.0001; Controls, *p* < 0.0001). We also found that the inverse relationship between baseline IL-10 and induced production of ΔTNFα and ΔIL-6 was similar among current, former and never smokers.

Given the known anti-inflammatory properties of IL-10 and its inverse relationship with cytokine responsiveness, we hypothesized that IL-10 would protect against respiratory symptoms. However, we found no association between chronic cough (*p* = 0.18), chronic phlegm (*p* = 0.31) and chronic bronchitis (*p* = 0.06) and baseline IL-10 levels using univariate logistic regression models (Table 2). Of those with respiratory symptoms (chronic cough, phlegm or bronchitis), approximately 35% were current smokers. Not surprising, smoking status was significantly associated with these chronic respiratory symptoms (*p* < 0.0001) in the adjusted analyses. In a sensitivity analysis, we stratified by smoking status and found that results were similar among never, former and current smokers. Also, similar results were observed when stratifying by COPD status.

**Table 2:** Association of IL-10 with Respiratory Symptoms

Disease Status	Ln IL-10 (pg/ml)					
	Chronic Cough		Chronic Phlegm		Chronic Bronchitis	
	n	Mean (SD)	n	Mean (SD)	n	Mean (SD)
No	398	4.36 (1.1)	393	4.34 (1.1)	453	4.36 (1.1)
Yes	205	4.23 (1.1)	207	4.24 (1.1)	143	4.17 (1.0)
p	0.18		0.31		0.06	
^a^p_adj_	0.33		0.79		0.18	
^a^OR_adj_ (95% CI)	0.92 (0.77–1.09)		0.98 (0.82–1.16)		0.87 (0.72–1.06)	

^a^Multivariable logistic regression models were adjusted for age, sex, BMI, race, education, smoking status and white blood cell count

Results of the multinominal regression are summarized in Table 3. Higher FEV_1_/FVC ratio (pairwise comparison of quartile 2, 3, 4 to quartile 1) was significantly associated with increased baseline IL-10 concentration. There was a 39% increase in baseline IL-10 concentration in quartile 2, 42% increase in IL-10 in quartile 3 and 32% increase in IL-10 in quartile 4. Covariates significantly and independently associated with higher FEV_1_/FVC included lower age and white blood cell count, and higher BMI. FEV_1_ and FVC were not significantly associated with baseline IL-10 concentration.

**Table 3:** Associations of IL-10 baseline concentration with quartiles of lung function

	FEV_1_/FVC			FEV_1_ (% predicted)			FVC (% predicted)		
	OR_adj_ (95% CI)	p	^a^p_adj_	OR_adj_ (95% CI)	p	*p_adj_	OR_adj_ (95% CI)	p	^a^p_adj_
Quartile 1	1.0 (referent)			1.0 (referent)			1.0 (referent)		
Quartile 2	1.39 (1.11–1.76)	0.002	0.005	1.05 (0.84–1.32)	0.78	0.19	1.00 (0.80–1.26)	0.70	0.98
Quartile 3	1.42 (1.12–1.81)	0.005	0.004	1.18 (0.94–1.49)	0.13	0.16	1.12 (0.89–1.41)	0.40	0.34
Quartile 4	1.32 (1.02–1.69)	0.014	0.033	1.17 (0.93–1.47)	0.21	0.66	0.98 (0.78–1.24)	0.70	0.87

^a^Multivariable nominal regression models were adjusted for age, sex, BMI, race, education, smoking status and white blood cell count. *n* = 601

## Discussion

In this study, we found that high systemic IL-10 levels were significantly associated with a protective response against organic dust-stimulated TNF-α and IL-6 hyper-responsiveness using an ex vivo whole blood assay. The inverse association between IL-10 levels and cytokine responsiveness was strongest for TNF-α. A significant relationship existed between FEV_1_/FVC ratio and IL-10 levels with lower IL-10 levels associated with lower FEV_1_/FVC ratio, even after adjusting for covariates. There were no associations observed for IL-10 levels and FEV_1_ or FVC percent predicted. Collectively, these studies support a potential role for IL-10 in modulating an inflammatory response in agriculture-exposed persons.

Agriculture workers are recurrently exposed to microbial component- and particulate matter-enriched organic dust aerosols. Whereas it is clear that exposure to these aerosols can lead to lung inflammation, it is not known how inflammatory responses are resolved in some individuals while others develop chronic lung disease. Thus, this study focused on IL-10 as a biomarker to potentially explain the susceptibility of agriculture-exposed persons to systemic and airway inflammatory disease. Within this cohort of agriculture-exposed persons conducted at the Veterans Administration Medical Center in the Midwestern United States, the majority were white males (96%), former smokers (58%), overweight or obese (85%), and had a history of exposure to livestock (87%) and crops (82%). Approximately one-third of the cohort reported chronic cough, chronic phlegm and/or chronic bronchitis. Importantly, IL-10 concentration was inversely related to the FEV_1_/FVC ratio, even after adjustment for covariates. We interpret our data to suggest that there is less airflow obstruction with high IL-10 levels. The relationship between serum IL-10 levels and airway IL-10 levels in this population is not known. Others have reported that COPD subjects with increased serum IL-10 levels also had increased sputum IL-10 as compared to controls [29]. However, a study of 72 coal workers with pneumoconiosis showed that lavage fluid IL-10 levels were significantly increased compared to controls, but serum IL-10 levels were not modulated [30]. Future studies should consider sputum and/or lavage fluid measurements for IL-10 in conjunction with serum IL-10 measurements. Moreover, the difference between localized versus systemic effects of IL-10 would be important to recognize in development of future strategies aimed at administration of IL-10. Low dose, systemic IL-10 administration has been well tolerated in phase I clinical trials in healthy subjects [31, 32], but it is also recognized that IL-10 could impede bacterial clearance leading to potential increase in infections [33].

Several studies have demonstrated that sputum IL-10 levels are suppressed in both healthy smokers and COPD subjects as compared to healthy, non-smoking controls [29, 34]. Zhang et al. further demonstrated that in a study of 94 COPD patients and 45 controls, both serum and sputum IL-10 levels were higher in healthy, non-smoking controls as compared to COPD patients and healthy smokers [35]. In a recent study, whereby serum cytokines and chemokines from 2123 subjects from COPDGene and 1117 subjects from SPIROMICS were analyzed, low serum IL-10 levels were associated with worse FEV_1_ (% predicted), but were not associated with progression of COPD or emphysema [36].

IL-10 is an anti-inflammatory and pro-resolving factor that can work to inhibit proinflammatory cytokines. In monocytes/macrophages, there has been shown a direct relationship whereby TNF-α activates IL-10, which in turn acts as a counter regulatory cytokine to inhibit TNF-α production. Consistent with this relationship, our study demonstrates for the first time in a human whole blood assay, that elevated levels of IL-10 appear to suppress TNF-α and IL-6 release following ODE exposure. This finding is important because it suggests that proinflammatory, hyper-responsiveness could be modulated by IL-10; moreover, it could suggest that IL-10 dysregulation might play a role in disease development. Future studies could investigate whether polymorphisms in the *IL-10* gene are associated with agriculture-related airway inflammatory disease. Others have shown that *IL-10* single nucleotide polymorphisms were associated with airway hyper-responsiveness, allergy, COPD, and high IL-10 levels [37–40]. It is also possible that IL-10 gene polymorphisms explain the variation in IL-10 levels amongst the study participants.

Organic dusts from confined animal feeding operations are complex mixtures of a wide variety and abundance of gram-positive and gram-negative bacterial components, proteases, and particulates that activate innate immune responses through Toll-like-receptor/MyD88 signaling pathways. In previous pilot work, we investigated and compared the ability of ODE, gram-negative lipopolysaccharide, and gram-positive peptidoglycan to stimulate TNF-α and IL-6 release in the whole blood assay of 10 healthy control subjects and 10 COPD subjects [41]. Stimulation of whole blood with ODE consistently and reproducibly stimulated TNF-α and IL-6 responses, which provided the rationale for the studies conducted in this current, large cohort of agriculture-exposed workers. Of note, we also found that the COPD subjects demonstrated increased TNF-α and IL-6 hyper-responsiveness as compared to healthy controls, whereas responses to lipopolysaccharide and peptidoglycan were inconsistent and low in the small pilot study [41].

There are limitations to this study. Only agriculture-exposed persons were enrolled by design; therefore, extrapolation of our findings to non-Veterans and non-agriculture exposed persons cannot be made. Another potential limitation of this study is that more than half of the subjects had a history of former or current cigarette smoke exposure, and although we adjusted for smoking and did a sensitivity analysis, it remains possible that the inverse relationship between baseline IL-10 and cytokine expression may not be specific to agricultural workers. To capture subjects with airway disease, the age of the cohort was also limited to persons aged 40–80 years, and subjects were predominately white males. The agriculture industry in the United States is changing demographically, but remains highly dominated by male workers. A recent study of dairy workers in Eastern Colorado demonstrated that these workers were largely non-smoking, Hispanic males aged 25–45 years with females accounting for 11% of the population [42]. Thus, it will be important in future studies to determine the role of IL-10 in different agriculture-exposed populations and geographic areas.

## Conclusions

In summary, in a large cohort of agriculture-exposed persons, a relationship was demonstrated between systemic IL-10 levels and proinflammatory cytokine response and lung function. There is growing evidence that IL-10 plays an important role in chronic airway inflammatory diseases, and that individual IL-10 differences might in part explain the predisposition to disease. It will be essential to define the cellular mechanistic pathway for the regulation of IL-10 in response to ODE. This pathway could be exploited to understand genetic differences and potentially modulated for therapeutic interventions to ultimately reduce disease burden in exposed persons.

## Abbreviations

BMI: Body mass index
COPD: Chronic obstructive pulmonary disease
IL: Interleukin
ODE: Organic dust extract
TNF-α: Tumor necrosis factor alpha
